# Is Watching TV Series an Adaptive Coping Strategy During the COVID-19 Pandemic? Insights From an Italian Community Sample

**DOI:** 10.3389/fpsyt.2021.599859

**Published:** 2021-04-21

**Authors:** Valentina Boursier, Alessandro Musetti, Francesca Gioia, Maèva Flayelle, Joël Billieux, Adriano Schimmenti

**Affiliations:** ^1^Department of Humanities, University of Naples Federico II, Naples, Italy; ^2^Department of Humanities, Social Sciences and Cultural Industries, University of Parma, Parma, Italy; ^3^Institute of Psychology, Faculty of Social and Political Sciences, University of Lausanne, Lausanne, Switzerland; ^4^Faculty of Human and Society Sciences, Kore University of Enna, Enna, Italy

**Keywords:** anxiety, binge-watching, watching TV series motives, COVID-19, coping strategies

## Abstract

Social distancing and lockdown due to the COVID-19 pandemic substantially impacted individuals' daily habits and well-being. Within such a context, digital technology may provide a welcome source of alternative forms of connection and entertainment. Indeed, streaming services showed a remarkable increase in membership subscriptions throughout the period considered. However, excessive involvement in watching TV series has recently become a subject of scholarly concern as it may represent an emerging form of addictive behavior with the features of what has been labeled as “binge-watching” (i.e., watching multiple episodes of TV series in a single session). The current study aimed to assess TV series watching behaviors and related motivations, as well as their relationships with depression, stress and anxiety, in a sample of Italian adults during the COVID-19 lockdown. Specifically, we aimed to explore which patterns of motivations and emotional states influenced either a high but healthy engagement in watching TV series, or promoted problematic and uncontrolled watching behavior under such circumstances. A total of 715 adults (*M* = 31.70, *SD* = 10.81; 71.5% female) from all over Italy were recruited (from 1st to 30th April 2020) through advertisements *via* social media platforms of Italian university communities and other online groups. Two multiple hierarchical regression analyses were performed with non-problematic and problematic TV series watching set as dependent variables. Results showed that people spent more time watching TV series during the pandemic lockdown, especially women who also reported higher levels of anxiety and stress than men. Moreover, both non-problematic (*R*^2^ = 0.56; *p* < 0.001) and problematic (*R*^2^ = 0.33; *p* < 0.001) TV series watching behaviors were equally induced by anxiety symptoms and escapism motivation, thereby suggesting that watching TV series during the COVID-19 lockdown probably served as a recovery strategy to face such a stressful situation. Finally, our findings also suggest that enrichment motives may protect from uncontrolled and potentially addictive watching behaviors. These findings, therefore, hold important implications, particularly for avoiding the over-pathologization of excessive involvement in online activities emerging as a result of specific distressing situations.

## Introduction

The recent COVID-19 pandemic has caused worldwide derangement. Governments imposed lockdown and measures of social distancing, ruling restrictions that highly affected individuals' daily routine and impacted on people's behaviors and psychological well-being (1–5). A wide body of international literature has thus investigated how the outbreak emergency has affected mental health (2, 6–10), forcing individuals to cope with uncertainty, fears, isolation and feelings of stress, anxiety and depression (3, 11, 12). A recent meta-analytic study indeed provided evidence of increased rates of depression (24%), anxiety (26%), post-traumatic stress symptoms (15%), and poor sleep quality (34%) in the general population following the Covid-19 outbreak (13). More specifically, Italy was the first European country to face the pandemic emergency, and recent studies involving Italian samples suggested that lonely as well as depressive individuals have been more likely to perceive the COVID-19 outbreak and related containment measures as distressful (14–17).

Notably, the use of digital technology has been recommended, as it provides alternative forms of connection and entertainment in an unprecedented period of social distancing and lockdown even though the effects of social media consumption in this specific circumstance need to be carefully addressed (18, 19), as recently showed (14, 20). From the 1st weeks of pandemic, media companies reported an exponential growth in media consumption by different types of users among generations, especially highlighting an increasing search for updated information among young and middle-aged individuals (21). More particularly, streaming service trends revealed a definite impact of COVID-19 quarantining with a sharp increase in membership subscriptions—for example, a 104% increase in Netflix subscribers and 633% in Disney Plus subscribers were observed between January and April 2020 at the worldwide level (22, 23). As regards Italy specifically, since March 2020, Netflix and the newcomer Disney Plus have recorded an increase of accesses of 332 and 290%, respectively (24).

Over the last decade, the concept of watching television has undergone a transition. Video-on-demand (VoD) services (e.g., Netflix, Amazon Prime, Rakuten) revolutionized viewing practices impacting on consumers' engagement (25). Indeed, these online streaming platforms offer permanently available programs (26), which implies that, unlike traditional TV viewers, VoD subscribers can watch TV series at their own convenience [i.e., *what, when, where* and *how* they want; (27)]. In this regard, watching multiple episodes of a TV series all in one go has become a very popular viewing pattern (28–30). Consequently, the implications of these changes in viewing practices are increasingly fueling the scientific debate (31–37) on the potential harmfulness of what has been labeled “binge-watching” (i.e., watching multiple episodes of TV series in a single session).

Binge-watching became better known in 2013, when the Oxford Dictionaries placed it in the Word of the Year shortlist (38). Rapidly, binge-watching has become a daily and widespread habit among TV series viewers as a part of a trend (27) reflecting a taste for immediate gratification (39) and/or a social tool to share opinions with friends, thereby reinforcing a sense of belongingness (40).

Previous research assessing binge-watching behaviors highlighted higher engagement among women (33, 41, 42) and young people (27, 43, 44). Moreover, scholars analyzed the relationships between psychopathological symptoms and binge-watching behaviors, pointing out a positive association between binge-watching and depression (27, 45) as well as anxiety (46). Thus, individuals experiencing negative affect and emotions might be more prone to engage in problematic binge-watching as a coping strategy (33, 47, 48). However, it was recently proposed that binge-watching induced by escapist motivations (i.e., motives related to coping with adverse life events or negative affect by immersing oneself in a TV series) can paradoxically contribute to recovery from stress (49).

In this regard, excessive involvement in watching TV series has recently become a matter of concern, leading scholars to debate on the differences between what reflects a non-problematic recreational activity (a healthy engagement or a “passion”) and what constitutes an excessive and uncontrolled form of behavior associated with negative consequences, functional impairment, and distress (34, 36, 47). Initial evidence indeed suggests that binge-watching may represent an emerging addictive behavior (50–52), which is reflected in individuals' loss of control over watching time (31, 50, 52, 53), impairment of day-to-day functioning (53), sleep quality (54, 55), and social relationships (53, 56).

Undoubtedly, the functionally impairing nature of the engagement has been evidenced as a critical dimension when considering problematic involvement in a specific behavior (57–59), and a key element that prevents from the risk of over-pathologizing everyday life activities (60). In this regard, particular attention should be paid to the motivations underlying binge-watching and its potential consequences (44, 48). Indeed, previous studies stated a wide range of motivations for engaging in watching TV series [e.g., social interaction, relaxation, escapism from reality, coping with stressful circumstances; (32, 42, 44, 47, 49, 61)]. Accordingly, relationships between various motives for watching TV series and unproblematic/problematic viewing behaviors (i.e., different levels of engagement or loss of control in binge-watching) is a key issue which needs to be considered (32). More specifically, individuals' engagement in watching TV series during the current pandemic deserves particular attention, as different motivations related to different levels of involvement in such activity might reflect adaptive or maladaptive responses to this unprecedented context.

The current study thus aimed to assess TV series viewing behaviors and related motivations, as well as their relationships with depression, stress and anxiety in a sample of Italian adults during the COVID-19 lockdown. Within this context, our particular aim was to explore which patterns of motivations and emotional states specifically influenced either a high but healthy involvement in watching TV series, or promoted a problematic and uncontrolled viewing behavior.

We not only hypothesized that psychopathological symptoms would affect TV series watching behaviors, but also that viewing motivations would particularly discriminate between healthy and problematic involvement in this activity. In particular, we predicted that coping/escapism motive could be related to both healthy and problematic involvement, whereas differences could be found concerning other motivations to watch TV series, such as those related to emotional enhancement, personal enrichment, and the fostering of social connection.

## Methods

### Participants and Procedure

A cross-sectional design was adopted during the COVID-19 pandemic emergency, covering the lockdown period in Italy that was declared on 9th March and was implemented across the entire country till 3rd May. A total of 715 adults from all over Italy participated in this study through an online survey system. Participants ranged in age from 18 to 72 (*M* = 31.70, *SD* = 10.81) and 71.5% of the sample were female (*n* = 511). Participants were recruited (from 1st to 30th April 2020) through advertisements in Italian university Web communities and other online groups (*via* social media platforms), which asked for dissemination among their members. There were no specific inclusion criteria, except being of legal age which, according to Italian law, is 18 years of age. The call for participation in the online study contained a website link for participants to click on to complete the questionnaire. Participation was voluntary, and confidentiality and anonymity were ensured. No course credits or remunerative rewards were given. Before filling out the survey, all of the participants were informed about the research aims and its scope, and the measures to be used in generating the data. The participants could withdraw from the study at any time. There were no missing responses because all of the questions were set as mandatory. The current study was approved by the University Federico II (Naples, Italy) Research Ethics Committee and was conducted according to the ethical guidelines for psychological research established by the Italian Psychological Association (AIP). Additional scales assessing individuals' social media use during the COVID-19 pandemic were also administered to this sample. Further findings of this broader research that are not directly relevant for the current study have been discussed elsewhere (14).

### Measures

#### Sociodemographic Information and Time Spent Watching TV Series

In this section, information was collected about gender, age, number of family members at home during the COVID-19 lockdown, and hours spent watching TV series per day before and during forced isolation due to COVID-19. A Δ score was calculated to reflect the difference between hours spent watching TV series during and before the COVID-19 lockdown.

#### Watching TV Series Engagement and Loss of Control

The extent of TV series watching involvement and problematic binge-watching was assessed using the Italian version of the 40-item Binge-Watching Engagement and Symptoms Questionnaire [BWESQ – (32); Italian version by (62)]. Relevant to the present research, only two subscales of the questionnaire were used in this study as reflecting adaptive vs. maladaptive TV series watching: *engagement* (e.g., “Watching TV series is one of my favorite hobbies.”) and *loss of control* (e.g., “I sometimes try not to spend so much time watching TV series, but I fail every time.”). Items are scored on a 4-point Likert scale ranging from 1 (*strongly disagree*) to 4 (*strongly agree*). Higher average score on each subscale indicates greater involvement or problematic binge-watching, respectively. The Cronbach's α values obtained in this study were 0.87 (*engagement*) and 0.82 (*loss of control*).

#### Psychopathological Symptoms

The Depression Anxiety Stress Scale [DASS-21 – (63); Italian version by (64)] was used to measure psychopathological symptoms. The DASS-21 is a 21-item self-report tool using a 4-point Likert scale ranging from 0 (*did not apply to me at all*) to 3 (*applied to me very much, or most of the time*), assessing depressive symptoms (e.g., “In the last 7 days, I felt no positive feelings”), anxiety symptoms (e.g., “In the last 7 days, I have had problems breathing”), and stress (e.g., “I found it hard to wind down”). Higher scores correspond to greater severity of psychopathological symptoms. The Cronbach's α values in this study were 0.90 (*depression*), 0.86 (*anxiety*), and 0.90 (*stress*).

#### Watching TV Series Motives

The Italian version of the Watching TV Series Motives Questionnaire [WTSMQ – (32); Italian version by (62)] was used to assess TV series watching motivations. It is a 22-item scale with four core dimensions: *coping/escapism* (e.g., “I watch TV series to escape reality and seek shelter in fictional worlds.”), *emotional enhancement* (e.g., “I watch TV series to be captivated and experience extraordinary adventures by proxy.”), *enrichment* (e.g., “I watch TV series to develop my personality and broaden my views.”), and *social* (e.g., “I watch TV series to relate to others more easily, because TV series give me something to talk about.”). Items are evaluated on a 4-point Likert scale ranging from 1 (*not at all*) to 4 (*to a great extent*), with a higher average score on each subscale indicating higher motivation for watching TV series. Cronbach's α values in this study were 0.87 (*coping/escapism*), 0.88 (*emotional enhancement*), 0.85 (*enrichment*), and 0.71 (*social*).

### Statistical Analyses

Descriptive statistics were computed for all of the study variables. Gender differences were examined through *t*-test and the magnitude of the differences was evaluated with effect sizes (Cohen's *d*). Pearson's *r* correlations were used to explore the associations between the variables. Finally, two multiple hierarchical regression analyses were performed. First, adaptive engagement in watching TV series (i.e., *engagement*) was set as the dependent variable, with sociodemographic characteristics (age, gender, and the number of family members at home during COVID-19 restrictions) and increased time spent watching TV series during COVID-19 restrictions (step 1), anxiety, depression, and stress symptom scores (step 2), as well as WTSMQ domain scores (step 3), set as predictors. Second, maladaptive engagement in watching TV series (i.e., *loss of control*) was set as the dependent variable, using the same set of predictors. A level of *p* < 0.05 was set as the level for statistical significance.

## Results

### Descriptive Statistics

Descriptive statistics are reported in Table 1 for both the full sample and differentiated by gender, along with the level of significance for gender differences. Participants reported 2.81 h/day spent watching TV series during the pandemic, with an increase of about one episode per day (0.84 h in average) in respect to their pre-COVID-19 watching habits. Females showed a higher increased amount of time watching TV series during the COVID-19 pandemic than males. Females also reported a higher extent of engagement in watching TV series, and higher levels of anxiety and stress symptoms. Males reported a higher motivation in bonding with others through watching TV series.

**Table 1:** Descriptive statistics and gender differences for all investigated variables.

	**Full sample (*****N*** **=** **715)**			**Males (*n* = 204)**	**Females (*n* = 511)**	***t*_**(713)**_**	***d***	**95% CI**
	**M (SD)**	**Observed range**	**Possible range**	**M (SD)**	**M (SD)**			
Δ h/day watching TV series during and before the COVID-19	0.84 (1.16)	−4 – 5	−24 – 24	0.70 (0.97)	0.89 (1.22)	−1.97^*^	0.17	[−0.38, 0.00]
BWESQ-Engagement	1.89 (0.67)	1 – 3.88	1 – 4	1.81 (0.59)	1.91 (0.70)	−2.03^*^	0.15	[−0.21, 0.00]
BWESQ-Loss of control	1.48 (0.53)	1 – 4	1 – 4	1.48 (0.50)	1.49 (0.53)	0.81	0.02	[−0.10, 0.07]
WTSMQ-Coping/Escapism	2.02 (0.68)	1 – 4	1 – 4	1.98 (0.62)	2.04 (0.71)	−1.17	0.09	[−0.17, 0.04]
WTSMQ-Enrichment	2.22 (0.83)	1 – 4	1 – 4	2.18 (0.79)	2.24 (0.84)	−0.83	0.07	[−0.19, 0.08]
WTSMQ-Emotional-enhancement	2.15 (0.81)	1 – 4	1 – 4	2.20 (0.79)	2.13 (0.83)	0.98	0.09	[−0.07, 0.20]
WTSMQ-Social	1.33 (0.49)	1 – 4	1 – 4	1.42 (0.56)	1.29 (0.46)	2.84^**^	0.25	[0.04, 0.21]
Depression	0.99 (0.75)	0 – 3	0 – 3	0.91 (0.70)	1.02 (0.77)	−1.86	0.15	[−0.24, 0.01]
Anxiety	0.69 (0.67)	0 – 3	0 – 3	0.59 (0.60)	0.73 (0.68)	−2.72^**^	0.22	[−0.24, −0.3]
Stress	1.36 (0.74)	0 – 3	0 – 3	1.23 (0.71)	1.41 (0.75)	−2.79^**^	0.25	[−0.29, −0.05]

* *p < 0.05;*

** *p < 0.01*.

Subsequently, the intercorrelations between the investigated variables were examined (see Table 2). More time spent watching TV series during the COVID-19 pandemic was significantly and positively associated with engagement in watching TV series scores and, to a lesser extent, also with loss of control over TV series watching, coping/escapism, enrichment, and emotional enhancement motives for watching TV series. No further associations were found between increased amount of time spent watching TV series during the COVID-19 pandemic and psychopathological symptoms domain scores.

**Table 2:** Pearson's *r* correlations between the variables.

	**2**	**3**	**4**	**5**	**6**	**7**	**8**	**9**	**10**	**11**	**12**	**13**
1. Gender	−0.01	0.06	−0.16^**^	−0.07	−0.01	−0.04	−0.03	0.04	0.11^**^	−0.07	−0.10^**^	−0.10^**^
2. Age	–	−0.25^**^	−0.22^**^	−0.33^**^	−0.25^**^	−0.37^**^	−0.43^**^	−0.34^**^	−0.20^**^	−0.25^**^	−0.18^**^	−0.21^**^
3. Number of family members at home		–	−0.01	0.04	0.07	0.03	0.03	0.00	0.10^**^	0.06	0.05	0.06
4. Hour per day spent watching TV series during COVID-19 pandemic			–	0.57^**^	0.33^**^	0.40^**^	0.35^**^	0.33^**^	0.09^*^	0.14^**^	0.13^**^	0.07
5. BWESQ-Engagement				–	0.61^**^	0.66^**^	0.61^**^	0.69^**^	0.37^**^	0.28^**^	0.24^**^	0.22^**^
6. BWESQ-Loss of control					–	0.52^**^	0.35^**^	0.47^**^	0.40^**^	0.31^**^	0.27^**^	0.24^**^
7. WTSMQ-Coping/Escapism						–	0.62^**^	0.74^**^	0.47^**^	0.48^**^	0.34^**^	0.39^**^
8. WTSMQ-Enrichment							–	0.69^**^	0.45^**^	0.32^**^	0.27^**^	0.27^**^
9. WTSMQ-Emotional-enhancement								–	0.45^**^	0.34^**^	0.22^**^	0.28^**^
10. WTSMQ-Social									–	0.26^**^	0.23^**^	0.19^**^
11. Depression										–	0.70^**^	0.74^**^
12. Anxiety											–	0.70^**^
13. Stress												–

* *p < 0.05*;

** *p < 0.01*.

As reported in Table 3, the first hierarchical regression analysis revealed that younger age (β = 0.33, *p* < 0.001), and increased amount of time spent watching TV series during the COVID-19 pandemic (β = 0.15, *p* < 0.001) positively predicted adaptive engagement in watching TV series (i.e., *engagement*) at Step 1. These control variables accounted for 14% of the variance. With the inclusion of psychopathological symptoms as predictors at Step 2, younger age (β = 0.28, *p* < 0.001), increased amount of time spent watching TV series during the COVID-19 pandemic (β = 0.15, *p* < 0.001), and depression symptoms (β = 0.17, *p* < 0.01) were positively associated with adaptive engagement in watching TV series. Finally, with the inclusion of motives for TV series watching at Step 3, the explained variance increased from 18 to 56%. Female gender (β = 0.07 *p* < 0.01), increased amount of time spent watching TV series during the COVID-19 pandemic (β = 0.08, *p* = 0.01), anxiety symptoms (β = 0.09, *p* = 0.02), and both coping/escapism (β = 0.26, *p* < 0.001), enrichment (β = 0.18, *p* < 0.001), and emotional enhancement (β = 0.37, *p* < 0.001) motivations for watching TV series had a significant positively predictive effect on non-problematic watching engagement.

**Table 3:** Regression: predictors of engagement in watching TV series during the COVID-19 pandemic.

	***F***	***R^**2**^***	***ΔR^**2**^***	***B***	**SE**	***t***	***P***
**Step 1**	28.87 (*p* < 0.001)	0.14	0.14				
Age				−0.21	0.00	−9.20	<0.001
Gender^*^				−0.09	0.05	−1.79	0.07
Number of family members at home				−0.02	0.02	−0.87	0.38
Δ h/day watching TV series during and before the COVID-19 pandemic				0.09	0.02	4.36	<0.001
**Step 2**	22.69 (*p* < 0.001)	0.18	0.04				
Age				−0.02	0.00	−7.68	<0.001
Gender				−0.07	0.05	−1.29	0.20
Number of family members at home				−0.02	0.02	−0.90	0.37
Δ h/day watching TV series during and before the COVID-19 pandemic				0.09	0.02	4.40	<0.001
Depression				0.15	0.05	3.04	<0.01
Anxiety				0.07	0.05	1.36	0.17
Stress				−0.01	0.05	−0.18	0.86
**Step 3**	80.70 (*p* < 0.001)	0.56	0.38				
Age				0.00	0.00	−0.93	0.35
Gender				−0.10	0.04	−2.63	<0.01
Number of family members at home				0.02	0.01	1.25	0.21
Δ h/day watching TV series during and before the COVID-19 pandemic				0.05	0.01	3.24	0.01
Depression				−0.04	0.04	−1.06	0.29
Anxiety				0.09	0.04	2.26	0.02
Stress				−0.07	0.04	−1.84	0.07
WTSMQ-Coping/Escapism				0.26	0.04	6.21	<0.001
WTSMQ- Enrichment				0.15	0.03	4.80	<0.001
WTSMQ- Emotional-enhancement				0.30	0.03	8.71	<0.001
WTSMQ- Social				0.01	0.04	0.38	0.71

* *Male coded as 1; female coded as 0*.

As reported in Table 4, the second hierarchical regression analysis revealed that younger age (β = 0.24, *p* < 0.001) and increased amount of time spent watching TV series during the COVID-19 pandemic (β = 0.08, *p* = 0.02) positively predicted maladaptive engagement over TV series watching (i.e., *loss of control*) at Step 1. These control variables accounted for 7% of the variance. At Step 2, younger age (β = 0.18, *p* < 0.001), increased amount of time spent watching TV series during the COVID-19 pandemic (β = 0.08, *p* = 0.02), depression symptoms (β = 0.22, *p* < 0.001), and anxiety symptoms (β = 0.11, *p* = 0.04) were positively related to loss of control over TV series watching during the COVID-19 pandemic. Finally, with the inclusion of motives for TV series watching at Step 3, the explained variance increased from 14 to 33%. Loss of control over TV series watching was positively predicted by anxiety symptoms (β = 0.12, *p* = 0.01), coping/escapism (β = 0.29, *p* < 0.001), emotional enhancement (β = 0.20, *p* < 0.001), and social (β = 0.17, *p* < 0.001) motivations for watching TV series, and negatively predicted by the enrichment motive for watching TV series (β = −0.10, *p* < 0.03).

**Table 4:** Regression: predictors of loss of control over TV series watching during the COVID-19 pandemic.

	***F***	***R^**2**^***	***ΔR^**2**^***	***B***	**SE**	***t***	***P***
**Step 1**	13.46 (*p* < 0.001)	0.07	0.07				
Age				−0.01	0.00	−6.52	<0.001
Gender				−0.01	0.04	−0.20	0.84
Number of family members at home				0.00	0.01	0.34	0.73
Δ h/day watching TV series during and before the COVID-19 pandemic				0.04	0.02	2.29	0.02
**Step 2**	16.83 (*p* < 0.001)	0.14	0.07				
Age				−0.01	0.00	−4.76	<0.001
Gender				0.02	0.04	0.44	0.66
Number of family members at home				0.00	0.01	0.35	0.73
Δ h/day watching TV series during and before the COVID-19 pandemic				0.04	0.02	2.28	0.02
Depression				0.15	0.04	3.83	<0.001
Anxiety				0.09	0.04	2.06	0.04
Stress				−0.02	0.04	−0.61	0.54
**Step 3**	31.72 (*p* < 0.001)	0.33	0.19				
Age				0.00	0.00	−1.47	0.14
Gender				−0.02	0.04	−0.65	0.52
Number of family members at home				0.01	0.01	0.99	0.32
Δ h/day watching TV series during and before the COVID-19 pandemic				0.02	0.01	1.10	0.27
Depression				0.03	0.04	0.81	0.42
Anxiety				0.09	0.04	2.45	0.01
Stress				−0.05	0.04	−1.35	0.18
WTSMQ-Coping/Escapism				0.23	0.04	5.71	<0.001
WTSMQ- Enrichment				0.06	0.03	−2.12	0.03
WTSMQ- Emotional-enhancement				0.13	0.03	3.95	<0.001
WTSMQ- Social				0.19	0.04	4.73	<0.001

*Male coded as 1; female coded as 0.

## Discussion

Recent literature has evidenced that the COVID-19 outbreak and related protective measures involved many risks to individuals' mental health (1–3, 5–8, 10, 12). In order to contribute to the ongoing debate on the psychological consequences of forced isolation due to the current pandemic, where the functionally impairing nature of one's engagement in web-related activities is an important issue to consider (65), the purpose of this study was to explore TV series watching behaviors (both from an adaptive and maladaptive perspective) and their underlying motivations, as well as their relationships with psychopathological symptoms during the COVID-19 lockdown in a sample of self-selected Italian adults.

The present findings firstly show that people spent more time watching TV series during the pandemic lockdown. In particular, consistent with the existing literature on binge-watching [e.g., (33, 41, 42)], women still proved more engaged in watching TV series during the COVID-19 emergency, while also showing higher levels of anxiety and stress than men. These results thus enter in resonance with previous data showing women's higher propensity to experience negative affect and low sense of mastery in negative circumstances, thus engaging in abstract and dysfunctional ruminative coping (66), and that female gender constitutes a risk factor for anxiety during the COVID-19 pandemic (67). Conversely, men were found to be more interested in bonding with others through watching TV series in such life circumstances. These findings can also be interpreted according to recent studies that showed gender inequality in experiencing the consequences of the COVID-19 restrictions, which differently impacted men's and women's lives as well as gender-role attitudes (e.g. work-family balance) (68–70).

As previously reported (46), the positive association between TV series watching involvement and anxiety—as also evidenced in the current sample—supports the idea that individuals experiencing unpleasant affect are more prone to use binge-watching as a coping strategy to get recovery from undesirable emotions, thus facing and regulating their negative moods (33, 47–49). Indeed, individuals' adaptive reaction to negative life circumstances might be facilitated by web-related activities, which can positively contribute to alleviate negative feelings, even though sometimes paving the way for problematic online engagement (71). It has also been demonstrated that while emotional enhancement and enrichment motivation for watching TV series is more strongly related to non-problematic watching behavior, coping-escapism motive is usually more strongly associated with problematic patterns of TV series watching (32, 62).

Interestingly, in the current sample loss of control over TV series watching was positively predicted by anxiety symptoms and coping/escapism motivation for watching TV series, but also by emotional enhancement and social drivers. It appears, therefore, that both “positive” and “negative” reinforcement motivations for watching behavior played a role in predicting the possibility of losing control while immersing oneself in TV series during the COVID-19 lockdown. In line with current neuroscientific research, it could be hypothesized that the pleasure deriving from the alleviation of pain combines with the pleasure deriving from positive emotions and relationships, thereby generating a complex rewarding process that may lead in some cases to a loss of control over the behavior (72). However, it is noteworthy that the enrichment motive was negatively associated with a maladaptive engagement in TV series watching. This might suggest that watching TV series for exploring new ideas, increasing knowledge, and enriching one's own perspective on contexts and situations may protect from uncontrolled and potentially addictive watching behaviors.

Non-problematic engagement in TV series watching was positively predicted by anxiety symptoms, coping/escapism, enrichment and emotional enhancement motivations for watching TV series, as well as by the increased amount of time spent watching TV series during the COVID-19 pandemic, and this especially for women. Therefore, besides the opposite effect of the enrichment motive, the results of both regression analyses do not highlight a clear distinction between non-problematic and problematic patterns of TV series watching behaviors, which were likely less dissociated from each other in the unprecedented context of the COVID-19 lockdown.

Be that as it may, the fact that both non-problematic and problematic TV series watching behaviors appear to be equally induced by anxiety and coping/escapism motivation — as hypothesized — centrally strengthens the notion that watching TV series during the COVID-19 lockdown probably served as a recovery strategy to face such a stressful situation. Furthermore, the current pattern of predictors once again reinforces that TV series watching activity, despite a high involvement, should not be considered as problematic *per se* as it might actually represent an effective coping strategy to deal with emotional distress by allowing viewers to find temporary shelter in the fictional world of a TV series, while experiencing pleasure, and fulfilling self-development and social needs during those times of isolation due to the COVID-19 pandemic.

We may reasonably assume, then, that TV series watching seemed to fuel viewers' minds with a different world, thereby distracting individuals from the pandemic distress. In this context, the possibility to watch TV series for personal enrichment might be key to prevent excessive watching behavior becoming a compulsive and uncontrollable habit (59), rather than a temporary and adequate coping strategy.

Limitations of this study need to be acknowledged. First, the current cross-sectional design limited the ability to formally test causative effects. Second, the well-known risk of biases due to the use of self-reported measures is also prevailing. Third, despite the representation of the entire Italian peninsula in our sample, the different geographic areas of Italy have been differently affected by the COVID-19-related health crisis, thereby limiting the generalizability of the present results. Finally, if these watching TV series behaviors and related motivations should be regarded as resulting from such specific circumstances, it would be worthwhile considering analyzing the lasting effects of the pandemic on individuals' viewing behaviors through longitudinal study designs. Moreover, differences and similarities between different cultural contexts might be also explored.

Despite these limitations, the present findings hold important implications, not only for binge-watching research, but also for avoiding the over-pathologization and stigmatization of excessive online behaviors that may emerge as a result of specific distressing situations and that, as recently showed (14, 20, 73), might instead be effective although attentively addressed in some limited periods for sustaining temporary recovery from psychological distress.

## Data Availability Statement

The raw data supporting the conclusions of this article will be made available by the authors, without undue reservation.

## Ethics Statement

The studies involving human participants were reviewed and approved by Ethical Committee of Psychological Research of the Department of Humanities of the University of Naples Federico II. The patients/participants provided their written informed consent to participate in this study.

## Author Contributions

VB was responsible for preparing the first draft of the article. AM analyzed the data. FG edited the manuscript. MF conceptually contributed to the development of the work. JB and AS critically revised the whole work for important intellectual content. All authors contributed to the study design, article, and approved the final version of the paper.

## Conflict of Interest

The authors declare that the research was conducted in the absence of any commercial or financial relationships that could be construed as a potential conflict of interest.
