# Corrigendum: Is Watching TV Series an Adaptive Coping Strategy During the COVID-19 Pandemic? Insights From an Italian Community Sample

**DOI:** 10.3389/fpsyt.2021.698404

**Published:** 2021-06-03

**Authors:** Valentina Boursier, Alessandro Musetti, Francesca Gioia, Maèva Flayelle, Joël Billieux, Adriano Schimmenti

**Affiliations:** ^1^Department of Humanities, University of Naples Federico II, Naples, Italy; ^2^Department of Humanities, Social Sciences and Cultural Industries, University of Parma, Parma, Italy; ^3^Institute of Psychology, Faculty of Social and Political Sciences, University of Lausanne, Lausanne, Switzerland; ^4^Faculty of Human and Society Sciences, Kore University of Enna, Enna, Italy

**Keywords:** anxiety, binge-watching, watching TV series motives, COVID-19, coping strategies

In the original article, there was a mistake in Table 2 as published. The corrected Table 2 appears below.

**Table 2:** Pearson's *r* correlations between the variables.

	**2**	**3**	**4**	**5**	**6**	**7**	**8**	**9**	**10**	**11**	**12**	**13**
1. Gender	−0.01	0.06	−0.16^**^	−0.07	−0.01	−0.04	−0.03	0.04	0.11^**^	−0.07	−0.10^**^	−0.10^**^
2. Age	–	−0.25^**^	−0.22^**^	−0.33^**^	−0.25^**^	−0.37^**^	−0.43^**^	−0.34^**^	−0.20^**^	−0.25^**^	−0.18^**^	−0.21^**^
3. Number of family members at home		–	−0.01	0.04	0.07	0.03	0.03	0.00	0.10^**^	0.06	0.05	0.06
4. Hour per day spent watching TV series during COVID-19 pandemic			–	0.57^**^	0.33^**^	0.40^**^	0.35^**^	0.33^**^	0.09^*^	0.14^**^	0.13^**^	0.07
5. BWESQ-Engagement				–	0.61^**^	0.66^**^	0.61^**^	0.69^**^	0.37^**^	0.28^**^	0.24^**^	0.22^**^
6. BWESQ-Loss of control					–	0.52^**^	0.35^**^	0.47^**^	0.40^**^	0.31^**^	0.27^**^	0.24^**^
7. WTSMQ-Coping/Escapism						–	0.62^**^	0.74^**^	0.47^**^	0.48^**^	0.34^**^	0.39^**^
8. WTSMQ-Enrichment							–	0.69^**^	0.45^**^	0.32^**^	0.27^**^	0.27^**^
9. WTSMQ-Emotional-enhancement								–	0.45^**^	0.34^**^	0.22^**^	0.28^**^
10. WTSMQ-Social									–	0.26^**^	0.23^**^	0.19^**^
11. Depression										–	0.70^**^	0.74^**^
12. Anxiety											–	0.70^**^
13. Stress												–

* *p < 0.05*;

** *p < 0.01*.

The authors apologize for this error and state that this does not change the scientific conclusions of the article in any way. The original article has been updated.

